# Lattice Thermal Conductivity of Mg_3_(Bi,Sb)_2_ Nanocomposites: A First-Principles Study

**DOI:** 10.3390/nano13222938

**Published:** 2023-11-13

**Authors:** Qing Peng, Xiaoze Yuan, Shuai Zhao, Xiao-Jia Chen

**Affiliations:** 1 School of Science, Harbin Institute of Technology, Shenzhen 518055, China; 2 The State Key Laboratory of Nonlinear Mechanics, Institute of Mechanics, Chinese Academy of Sciences, Beijing 100190, China; 3 Guangdong Aerospace Research Academy, Guangzhou 511458, China; 4 Department of Modern Mechanics, University of Science and Technology of China, Hefei 230026, China

**Keywords:** Mg_3_(Bi_x_Sb_1−x_)_2_, lattice thermal conductivity, first-principles calculations

## Abstract

Mg_3_(Bi_x_Sb_1−x_)_2_ (0 ≤ x ≤ 1) nanocomposites are a highly appealing class of thermoelectric materials that hold great potential for solid-state cooling applications. Tuning of the lattice thermal conductivity is crucial for improving the thermoelectric properties of these materials. Hereby, we investigated the lattice thermal conductivity of Mg_3_(Bi_x_Sb_1−x_)_2_ nanocomposites with varying Bi content (x = 0.0, 0.25, 0.5, 0.75, and 1.0) using first-principles calculations. This study reveals that the lattice thermal conductivity follows a classical inverse temperature-dependent relationship. There is a significant decrease in the lattice thermal conductivity when the Bi content increases from 0 to 0.25 or decreases from 1.0 to 0.75 at 300 K. In contrast, when the Bi content increases from 0.25 to 0.75, the lattice thermal conductivity experiences a gradual decrease and reaches a plateau. For the nanohybrids (x = 0.25, 0.5, and 0.75), the distribution patterns of the phonon group velocity and phonon lifetime are similar, with consistent distribution intervals. Consequently, the change in lattice thermal conductivity is not pronounced. However, the phonon group speed and phonon lifetime are generally lower compared to those of the pristine components with x = 0 and x = 1.0. Our results suggest that the lattice thermal conductivity is sensitive to impurities but not to concentrations. This research provides valuable theoretical insights for adjusting the lattice thermal conductivity of Mg_3_(Bi_x_Sb_1−x_)_2_ nanocomposites.

## 1. Introduction

Thermoelectric (TE) materials can convert thermal energy into electricity, making them a potential solution to the current energy crisis [1,2,3,4,5,6,7,8,9]. Among these materials, Mg_3_(Bi_x_Sb_1−x_)_2_ is the most promising candidate for TE applications near room temperature (RT) due to its lower cost than the commercially available Bi_2_Te_3−x_Se_x_ [10,11,12]. As a result, it has received considerable attention [13,14,15,16,17,18,19,20,21,22,23,24,25,26]. Researchers are constantly working to improve the thermoelectric performance of these materials. The thermoelectric figure of merit, ZT, is typically used to evaluate performance, which is defined as $zT=S^{2}\sigma T/(\kappa_{l}+\kappa_{e})$. The parameters T, S, σ, $\kappa_{l}$, and $\kappa_{e}$ represent the Kelvin temperature, Seebeck coefficient, electrical conductivity, lattice thermal conductivity, and electronic thermal conductivity, respectively [27]. Achieving a high thermoelectric figure of merit requires a high Seebeck coefficient (S), high electrical conductivity (σ), and low thermal conductivity (κ) [28]. Yet, achieving this is challenging due to the complicated interplay among these parameters. It is believed that reducing the lattice thermal conductivity is crucial for achieving high TE performance.

There are currently many methods available to reduce the thermal conductivity of thermoelectric materials. Biswas et al. achieved the maximum reduction in the lattice thermal conductivity of PbTe thermoelectric materials via considering scattering sources on all relevant length scales in a hierarchical manner, ranging from atomic-scale lattice disorder and nanoscale endotaxial precipitates to mesoscale grain boundaries [29]. Nakamura et al. overcame the amorphous limit of thermal conductivity in Si thermoelectric materials through constructing a Si nanostructure with “well-controlled nanoscale-shaped interfaces” and oriented nanocrystals (NCs), resulting in a significant reduction in thermal conductivity [30]. Zhang et al. achieved a reduction in thermal conductivity and improved thermoelectric performance through doping Sb in Mg_2_(Si,Sn) compounds [31]. Mo et al. demonstrated that doping a small amount of Se into Mg_3_Bi_1.4_Sb_0.6_ can effectively reduce the thermal conductivity of the system [32]. Knura et al. found that alloying PbTe with SnTe leads to a significant reduction in lattice thermal conductivity to values below approximately 1.0 W m^−1^ K^−1^ across a wide range of compositions (from x = 0.25 to x = 0.80) [33]. Here, we focus on the effect of alloying Mg_3_Bi_2_ with Mg_3_Sb_2_ on the lattice thermal conductivity of the Mg_3_(Sb,Bi)_2_ system.

The lattice thermal conductivity of Mg_3_X_2_ (X = Bi, Sb) has been thoroughly researched. Mg_3_X_2_ (X = Bi and Sb) has an uncommonly low lattice thermal conductivity that can be attributed to the notable softening and flattening of low-energy transverse acoustic phonons [15,34,35]. Zhang et al. offered new insights into the low lattice thermal conductivity of Mg_3_Bi_2_ through relating the pronounced phonon anharmonicity to the asymmetric nature of Bismuth’s 6s lone-pair electrons [15]. Contrary to the anticipated T^−1^ temperature-dependent $\kappa_{l}$ at high temperatures, Zhu et al. discovered that Mg_3_Sb_2_ exhibits a feeble temperature dependence of $\kappa_{l}$ following a power law of T^−0.48^ from theory and T^−0.57^ from experimental measurements. This weak dependence can be traced back to the stiffening of low-lying phonons and diminished anharmonicity at high temperatures, as indicated by the authors [36]. Mg_3_Sb_2_ and Mg_3_Bi_2_ can be combined in different stoichiometric ratios [37,38,39,40]. The combination of these compounds has been found to lower lattice thermal conductivity [41]. Nonetheless, it is still a major challenge to synthesize ternary Mg_3_(Bi,Sb)_2_ with a controllable Bi/Sb ratio [42].

Computational simulations are crucial for studying the thermal properties of Mg_3_(Bi,Sb)_2_. Various first-principles-based software, including phono3py [43] and ShengBTE [44], have been used to explore phonon transport phenomena. The lattice thermal conductivity of Mg_3_(Bi,Sb)_2_ has been studied to a limited extent using first-principles calculations, unlike that of Mg_3_X_2_ (X = Bi, Sb), due to its computational cost. Additionally, machine learning potentials have emerged as an alternative approach to expedite research on heat transport [45,46,47,48]. hiPhive, developed by Eriksson et al., efficiently extracts high-order force constants from density functional theory calculations [49,50]. Yang et al. utilized a machine learning potential function based on dual adaptive sampling to investigate the lattice thermal conductivity of Mg_3_Sb_2_, and obtained results consistent with the experimental data [51]. Ouyang and colleagues conducted a molecular dynamics (MD) exploration of the thermal properties of Mg_3_(Bi_x_Sb_1−x_)_2_ using a moment tensor potential (MTP) model [52,53]. They developed an MTP model, which was based on machine learning (ML). Their predictions of changes in thermal conductivity with varying solution concentrations were generated using molecular dynamics simulations based on ML-interatomic potential (MLIAP) [54].

In this research, we analyze the lattice thermal conductivity of Mg_3_(Bi_x_Sb_1−x_)_2_ (0 ≤ x ≤ 1) utilizing first-principles calculations. Initially, we assess the crystal structure, thermodynamic stability, and dynamic stability of Mg_3_(Bi_x_Sb_1−x_)_2_ at different Bi contents (x = 0.0, 0.25, 0.5, 0.75, and 1.0). Subsequently, we examine the heat capacity, phonon group velocity, and phonon lifetime of Mg_3_(Bi_x_Sb_1−x_)_2_ to identify the factors that affect the changes in lattice thermal conductivity in the presence of various Bi contents.

## 2. Methods

The unit cell structure of Mg_3_Bi_2_ and Mg_3_Sb_2_ comprises five atoms. To investigate alternative structures, we expanded the Mg_3_Bi_2_ unit cell to create a 1 × 1 × 2 supercell. In this supercell, Sb atoms substitute Bi atoms, yielding three nanohybrid structures: Mg_3_Bi_1.5_Sb_0.5_, Mg_3_BiSb, and Mg_3_Bi_0.5_Sb_1.5_. We used first-principles calculations with VASP [55,56] to determine the lowest-energy structures for each composition. To compute the phonon spectrum, we utilized Phonopy [57,58,59] via the finite-displacement supercell approach. In the finite-displacement supercell approach, the first-principles calculation is utilized to acquire atomic forces in the supercell crystal structure model. Force constants were computed from an adequate number of supercells with distinct sets of displacements and corresponding forces were obtained using the first-principles calculation. For Mg_3_Bi_2_ and Mg_3_Sb_2_, 3 × 3 × 2 supercells were utilized, while 3 × 3 × 1 supercells were employed for Mg_3_Bi_1.5_Sb_0.5_, Mg_3_BiSb, and Mg_3_Bi_0.5_Sb_1.5_.

The thermal conductivity $\kappa_{l}$ is closely related to heat capacity, phonon group velocity, and phonon lifetime, as shown in Equation (1):

(1) $$\kappa_{l}=\frac{1}{NV_{0}}\sum_{\lambda }C_{\lambda }v_{\lambda }^{2}\tau_{\lambda }$$

where λ represents the phonon mode, N is the total number of q points used to sample the Brillouin zone, $V_{0}$ stands for the volume of a unit cell, and $C_{\lambda }$, $v_{\lambda }$, and $\tau_{\lambda }$ indicate the specific heat capacity, group velocity, and phonon lifetime, respectively. The $C_{\lambda }$ values were determined via analyzing the phonon density of states using the Bose–Einstein distribution. On the other hand, the $v_{\lambda }$ values were obtained via calculating the gradient of the phonon dispersion relation. To account for various phonon scattering mechanisms, the phonon relaxation time $\tau_{\lambda }$ can be divided into three contributions: phonon–phonon scattering, phonon–grain boundary scattering, and phonon–defect scattering. This division is performed using Matthiessen’s rule [60]. In the current study, our main focus is on phonon–phonon scattering. We calculated the phonon relaxation time for this type of scattering using the harmonic and third-order anharmonic force constants obtained from density functional theory (DFT) calculations. To solve the phonon Boltzmann transport equation (BTE) [61] and calculate the lattice thermal conductivity, we employed the phono3py framework within the DFT framework. Phono3py is a tool that allows us to compute properties related to phonon–phonon interactions. At each step, the users invoked phono3py with at least the unit cell and the supercell matrix. In the first step, we generated supercells and sets of atomic displacements. These sets of displacements are referred to as “displacement datasets”. We constructed supercells using these displacements. Next, we calculated the forces of the supercell models using the VASP software, which we refer to as “force sets”. In the second step, we computed the second- and third-order force constants (fc2 and fc3) from the displacement datasets and force sets obtained in the first step. Finally, in the third step, we utilized the force constants obtained in the second step to calculate various properties, such as the lattice thermal conductivity, specific heat capacity, phonon group velocity, phonon lifetime, and mode-Grüneisen parameters. Due to the computationally intensive nature of lattice thermal conductivity calculations, particularly for larger unit cells, we used relatively smaller supercells in our computations. We utilized a 2 × 2 × 1 supercell for Mg_3_Bi_1.5_Sb_0.5_, Mg_3_BiSb, and Mg_3_Bi_0.5_Sb_1.5_. Considering that the original structure of Mg_3_(Bi_x_Sb_1−x_)_2_ (x = 0.5, 1.0, and 1.5) is based on the 1 × 1 × 2 supercell of Mg_3_Bi_2_, the lattice thermal conductivity was calculated based on the 2 × 2 × 1 supercell. Therefore, for Mg_3_Sb_2_ and Mg_3_Bi_2_, we used a 2 × 2 × 2 supercell when calculating the lattice thermal conductivity. In this way, the calculation of the lattice thermal conductivity of Mg_3_(Bi_x_Sb_1−x_)_2_(0 ≤ x ≤ 1) can be kept in the same dimension, and the number of atoms in the supercell is 40. For the purpose of comparing the impact of cell size on the calculation of lattice thermal conductivity, we utilized Mg_3_Sb_2_ as an example and calculated the lattice thermal conductivity of a larger 4 × 4 × 3 supercell using the hiPhive software in combination with phono3py.

The VASP calculation parameters were chosen as follows. The exchange and correlation functions were approximated using the general gradient approximation with Perdew–Burke–Ernzerhof parameterization (GGA-PBE) [62]. The energy cutoff for the plane-wave basis expansion was set to 450 eV. *k*-mesh were produced using VASPKIT [63] in the Gamma scheme at a density of 2π×0.03 Å^−1^. For geometry optimization, a convergence criterion of 1 × 10^−5^ eV in energy and 1 × 10^−4^ eV/Å in force was applied. For the calculations of the phonon spectrum and lattice thermal conductivity, a convergence criterion of 1 × 10^−8^ eV in energy was utilized.

## 3. Results and Discussions

**Figure 1:**
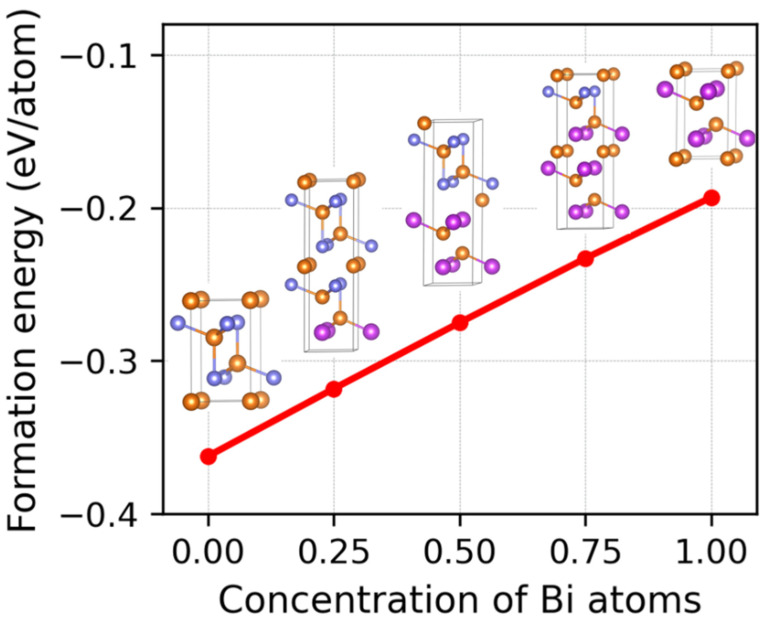
The formation energies and crystal structures of Mg_3_(Bi_x_Sb_1−x_)_2_ (0 ≤ x ≤ 1).

The formation energies and crystal structures of Mg_3_(Bi_x_Sb_1−x_)_2_ (0 ≤ x ≤ 1) are presented in Figure 1. The formation energies of Mg_3_(Bi_x_Sb_1−x_)_2_ were calculated using Equation (2):

(2) $$E_{form}=\left[E_{unitcell}^{{Mg}_{3}{\left({Bi}_{x}{Sb}_{1-x}\right)}_{2}}-3E_{form}^{Mg}-2xE_{form}^{Bi}-2(1-x)E_{form}^{Sb}\right]/5$$

In this equation, $E_{unitcell}^{{Mg}_{3}{\left({Bi}_{x}{Sb}_{1-x}\right)}_{2}}$ represents the total energy of a single chemical formula Mg_3_(Bi_x_Sb_1−x_)_2_, and $E_{form}^{Mg}$, $E_{form}^{Bi}$, $E_{form}^{Sb}$ denote the single atomic energies of Mg, Bi, and Sb pure constituents, respectively. The structures of Mg, Bi, and Sb were retrieved from the Material Project database [64] and have space groups of P6_3_/mmc, $R\bar{3}m,$ and $R\bar{3}m$, respectively. The single atomic energies of the pure constituents Mg, Bi, and Sb are −1.508 eV/atom, −3.9 eV/atom, and −4.13 eV/atom, respectively. As demonstrated in Figure 1, the formation energies of Mg_3_(Bi_x_Sb_1−x_)_2_ are negative, indicating thermodynamic favorability.

**Figure 2:**
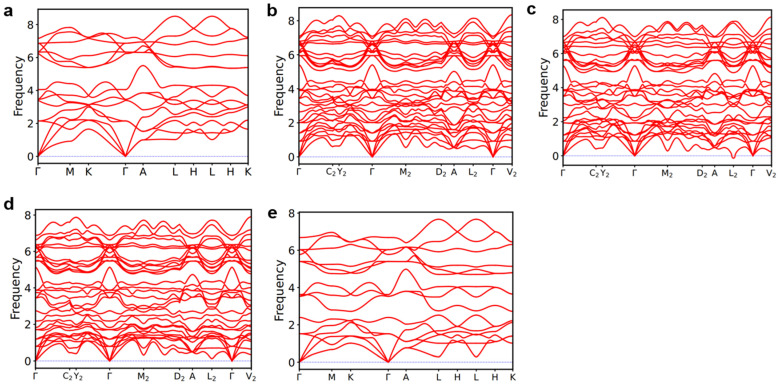
The phonon spectra of Mg_3_(Bi_x_Sb_1−x_)_2_ for (**a**) x = 0.0; (**b**) x = 0.25; (**c**) x = 0.5; (**d**) x = 0.75; (**e**) x = 1.0.

Additionally, the formation energy increases linearly as the Bi content increases. Figure 2 illustrates the phonon spectra of Mg_3_(Bi_x_Sb_1−x_)_2_. With the exception of the negligible imaginary frequency observed for Mg_3_BiSb at the L_2_ point, the remaining structures exhibit no imaginary frequencies, indicating dynamic stability. Moreover, the absence of a phonon bandgap facilitates a plethora of phonon–phonon scattering processes.

The Zintl compounds Mg_3_Sb_2_ and Mg_3_Bi_2_ share a trigonal CaAl_2_Si_2_-type structure and belong to the space group $P\bar{3}m1$ [65]. The polyanions (Mg_2_X_2_)^2−^ (X = Bi, Sb), in which Mg is tetrahedrally coordinated, form covalently bonded layers. These layers are ionically bonded with the octahedrally coordinated Mg^2+^ cation layers, forming the overall framework. Substituting Bi atoms with Sb results in a decrease in system symmetry. As the concentration of Bi increases, the gradual substitution of Bi atoms by Sb atoms occurs in a layer-by-layer manner, resulting in an expansion of the unit cell volume. Figure S1 in the Supplementary Information depicts the average lengths of Mg-X bonds in Mg_3_(Bi_x_Sb_1−x_)_2_ (0 ≤ x ≤ 1). In the (Mg_2_X_2_)^2−^ network, the vertical Mg-X bonds are longer than the three symmetry-equivalent tilted Mg-X bonds. In addition, the ionic M-X bonds are longer than the covalent M-X bonds. As the Bi content increases, so does the average bond length between M and X, suggesting a diminished bonding ability between Mg-X and the increased Bi content.

**Figure 3:**
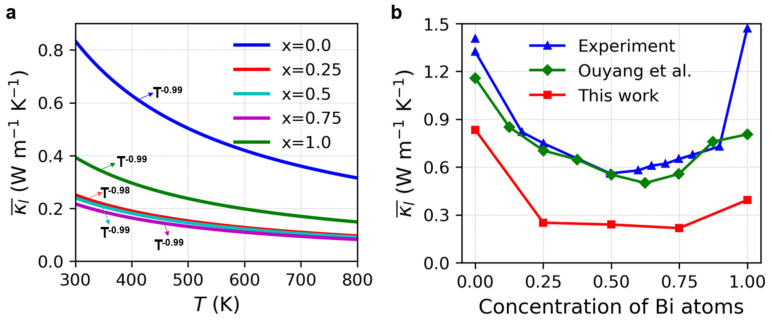
(**a**) Average lattice thermal conductivity of Mg_3_(Bi_x_Sb_1−x_)_2_ (0 ≤ x ≤ 1) at various temperatures; (**b**) Average lattice thermal conductivity of Mg_3_(Bi_x_Sb_1−x_)_2_ at 300 K for different Bi concentration compared with the experiment [41] and calculation by Ouyang et al. [54].

**Figure 4:**
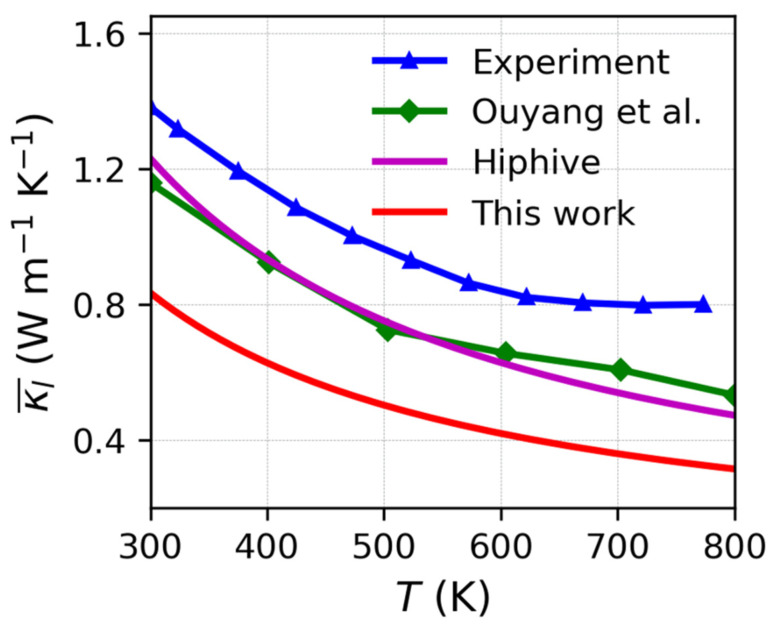
Average lattice thermal conductivity of Mg_3_Sb_2_ at various temperatures in 2 × 2 × 2 supercells compared with the hiPhive calculation in 4 × 4 × 3 supercells, experiment [66], and the MD calculation by Ouyang et al. in 2 × 2 × 30 supercells [54].

Figure 3a presents the average lattice thermal conductivities of Mg_3_(Bi_x_Sb_1−x_)_2_ (0 ≤ x ≤ 1) obtained from the BTE utilizing second- and third-order force constants calculated using DFT. These conductivities demonstrate a classical temperature-dependent relationship of T^−1^ instead of a weak temperature-dependent $\kappa_{l}$. It is observed that the order of lattice thermal conductivity is as follows: Mg_3_Sb_2_ (x = 0) > Mg_3_Bi_2_ (x = 1) > Mg_3_(Bi_x_Sb_1−x_)_2_ (x= 0.25, 0.5, 0.75). Alloying Mg_3_Bi_2_ with Mg_3_Sb_2_ significantly reduces the lattice thermal conductivity. We also analyzed the lattice thermal conductivity at 300 K for various Bi contents and compared it with the previous literature [41,54]. Notably, there were marked reductions in the lattice thermal conductivity as the Bi content increased from 0 to 0.25 or decreased from 1.0 to 0.75. However, as the Bi content increased from 0.25 to 0.75, the lattice thermal conductivity showed a slight decrease, indicative of a plateau. Thus, adjusting the Bi content within the range of x = 0.25 to 0.75 may have a negligible effect on the lattice thermal conductivity. Ouyang and colleagues employed ML-IAP to compute the lattice thermal conductivity of the Mg_3_(Bi_x_Sb_1−x_)_2_ alloy at different alloying concentrations for MCMD structures at 300 K [54]. Our findings are in qualitative concurrence with the experiment [41] and calculation by Ouyang et al., albeit our computed lattice thermal conductivities are comparatively lower. This difference could be due to the reduced size of the supercell employed in the phono3py computations. Due to the significant computational resources needed for larger supercells when calculating the lattice thermal conductivity, we selected Mg_3_Sb_2_ as an illustrative example. We assessed the lattice thermal conductivity in a 4 × 4 × 3 supercell using the hiPhive software and the results, demonstrated in Figure 4, generally indicate that the thermal conductivity acquired from the 4 × 4 × 3 supercell using hiPhive exceeds that of the 2 × 2 × 2 supercell calculated using phono3py. Furthermore, the outcomes generated using hiPhive coincide with the calculations carried out by Ouyang et al. [54], even though both sets of calculations produce marginally lower values in comparison to the experimental data [66].

**Figure 5:**
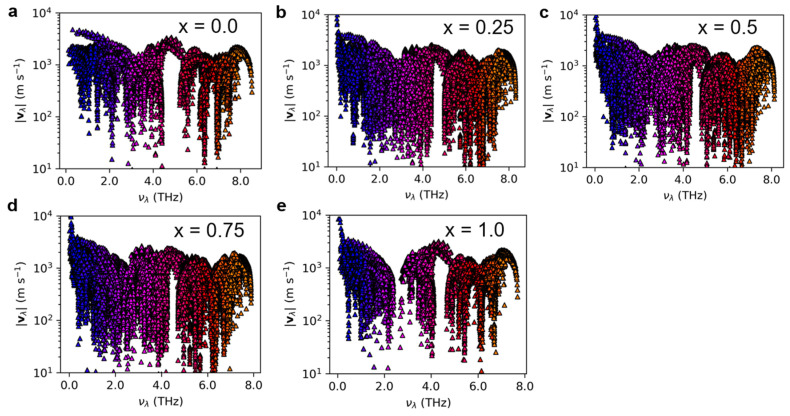
The phonon group velocity of Mg_3_(Bi_x_Sb_1−x_)_2_ for (**a**) x = 0.0; (**b**) x = 0.25; (**c**) x = 0.5; (**d**) x = 0.75; (**e**) x = 1.0.

**Figure 6:**
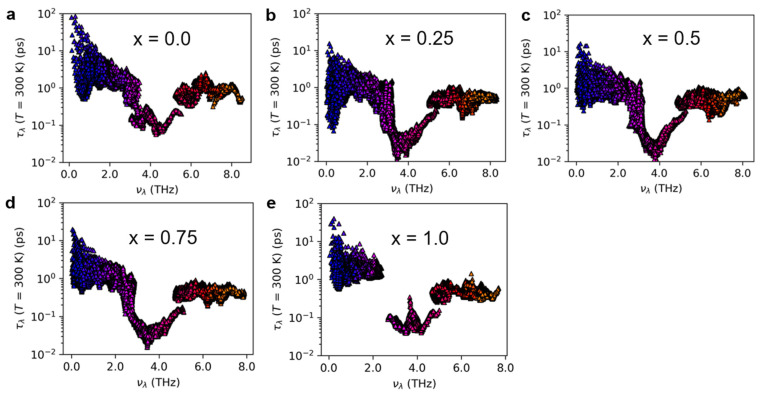
The phonon lifetime of Mg_3_(Bi_x_Sb_1−x_)_2_ at 300 K for (**a**) x = 0.0; (**b**) x = 0.25; (**c**) x = 0.5; (**d**) x = 0.75; (**e**) x = 1.0.

**Figure 7:**
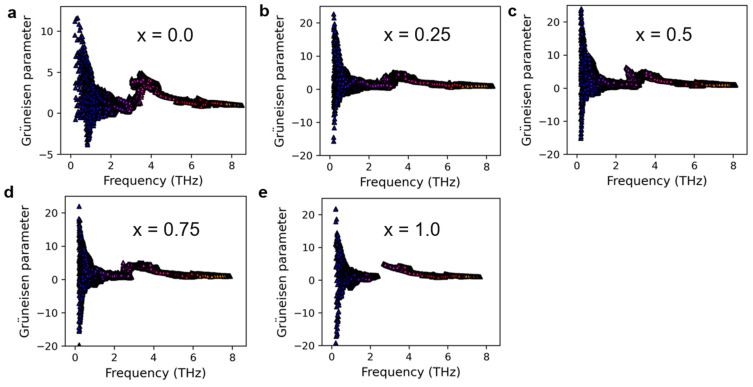
The mode-Grüneisen parameters of Mg_3_(Bi_x_Sb_1−x_)_2_ for (**a**) x = 0.0; (**b**) x = 0.25; (**c**) x = 0.5; (**d**) x = 0.75; (**e**) x = 1.0.

Figure S2 in the Supplementary Information presents the heat capacity of Mg_3_(Bi_x_Sb_1−x_)_2_ (0 ≤ x ≤ 1) at 300 K. The difference in thermal conductivity mainly arises from the phonon group velocity and phonon lifetime rather than the heat capacity, which exhibits only a small discrepancy. Figure 5 and Figure 6 display the phonon group velocity and phonon lifetime, respectively, of Mg_3_(Bi_x_Sb_1−x_)_2_. The phonon group velocity distribution range remains similar when x equals 0.25, 0.5, and 0.75. However, the overall group velocity is lower when compared to x equals 0 and 1.0, with the exception of a few higher group velocities in the low-frequency region. As shown in Figure 5, for x equals 0.25, 0.5, and 0.75, the proportion of phonon group speeds below 100 m/s increases significantly. Although the range of phonon group velocity distributions in Mg_3_Sb_2_ and Mg_3_Bi_2_ is consistent, the high-frequency region reveals that Mg_3_Bi_2_ has a significantly lower phonon lifetime than Mg_3_Sb_2_. Consequently, the lattice thermal conductivity of Mg_3_Bi_2_ is lower than that of Mg_3_Sb_2_. For x = 0.25, 0.5, and 0.75, the lifetime distribution pattern of phonons in Mg_3_(Bi_x_Sb_1−x_)_2_ is similar, with the distribution interval falling within the same range. Nonetheless, the overall distribution is lower in comparison to x = 0 and x = 1.0. According to Figure 6, there is a significant increase in the proportion of phonon lifetimes below 1.0 ps at x = 0.25, 0.5, and 0.75. We further examined the mode-Grüneisen parameters of Mg_3_(Bi_x_Sb_1−x_)_2_ (0 ≤ x ≤ 1), as illustrated in Figure 7. We found that compared to Mg_3_Sb_2_ and Mg_3_Bi_2_, there is a significant increase in the proportion of regions in the low-frequency range of Mg_3_(Bi_x_Sb_1−x_)_2_ with absolute values greater than 10 when x = 0.25, 0.5, and 0.75. This indicates that Mg_3_(Bi_x_Sb_1−x_)_2_ (x = 0.25, 0.5, and 0.75) exhibits higher anharmonicity. Therefore, as the Bi content increases from 0 to 0.25 or decreases from 1 to 0.75, the lattice thermal conductivity of Mg_3_(Bi_x_Sb_1−x_)_2_ decreases considerably. However, when the concentration changes from 0.25 to 0.75, the change in lattice thermal conductivity reaches a plateau.

## 4. Conclusions

We conducted a comprehensive investigation into the lattice thermal conductivity of Mg_3_(Bi_x_Sb_1−x_)_2_ (0 ≤ x ≤ 1) using DFT calculations. The average lengths of the Mg-X ionic bonds (d1), vertical Mg-X covalent bonds (d2), and three symmetry-equivalent tilted covalent Mg-X bonds (d3) all increase as the Bi content increases. The formation energies of Mg_3_(Bi_x_Sb_1−x_)_2_ are all negative, indicating that their crystal structures are thermodynamically stable. The dynamic stability of these structures is confirmed through the absence of imaginary frequencies in their phonon spectra.

The results from the phono3py calculations reveal that the lattice thermal conductivity of Mg_3_(Bi_x_Sb_1−x_)_2_ (0 ≤ x ≤ 1) with different Bi contents follows a descending order as Mg_3_Sb_2_ > Mg_3_Bi_2_ > Mg_3_(Bi_x_Sb_1−x_)_2_ (x = 0.25, 0.5, 0.75). At 300 K, the lattice thermal conductivity of Mg_3_(Bi_x_Sb_1−x_)_2_ is higher at both the upper and lower bounds of the composition range. The effect of Bi content on the lattice thermal conductivity is negligible for x in the range between 0.25 and 0.75. This trend is directly related to the phonon group velocity and phonon lifetime. The phonon lifetime of Mg_3_Sb_2_ is higher than that of Mg_3_Bi_2_. When x = 0 and x = 1.0, the phonon group velocity and phonon lifetime of Mg_3_(Bi_x_Sb_1−x_)_2_ are significantly higher compared to the values when x = 0.25, 0.5, and 0.75. When the Bi content increases from 0.25 to 0.75, there is little change in the distribution patterns for the phonon group velocity and phonon lifetime. Despite the increase in Bi content, the distribution range remains consistent, leading to negligible changes in the lattice thermal conductivity.

It is worth noting that the current calculation of lattice thermal conductivity for Mg_3_(Bi_x_Sb_1−x_)_2_ is based on a relatively small supercell with just 40 atoms. While a qualitative agreement between the calculated results and the experimental results is observed, some excluded phonons with shorter wavelengths lead to lower results due to the simulation box’s limitation. Our research contributes not only to the comprehension of thermal transport in Mg_3_(Bi_x_Sb_1−x_)_2_ but also to the optimization of its thermal conductivity through experimentation.

## Data Availability

Data available on request from the authors upon reasonable request.

## Supplementary Materials

The following supporting information can be downloaded at https://www.mdpi.com/article/10.3390/nano13222938/s1, Figure S1. (a) Crystal structure model of Mg_3_X_2_ (X = Sb, Bi); (b) The average lengths of Mg-X ionic bonds ($\bar{d_{1}}$), vertical Mg-X covalent bonds ($\bar{d_{2}}$), and three symmetry-equivalent tilted covalent Mg-X bonds ($\bar{d_{3}}$); Figure S2. The heat capacity of Mg_3_(Bi_x_Sb_1−x_)_2_ (0 ≤ x ≤ 1) at 300 K.

## References

[1] He J., Tritt T.M. (2017). Advances in thermoelectric materials research: Looking back and moving forward. Science.

[2] Mao J., Liu Z., Zhou J., Zhu H., Zhang Q., Chen G., Ren Z. (2018). Advances in thermoelectrics. Adv. Phys..

[3] Yang L., Chen Z.-G., Dargusch M.S., Zou J. (2018). High performance thermoelectric materials: Progress and their applications. Adv. Energy Mater..

[4] Tritt T.M. (2011). Thermoelectric phenomena, materials, and applications. Annu. Rev. Mater. Sci..

[5] Twaha S., Zhu J., Yan Y., Li B. (2016). A comprehensive review of thermoelectric technology: Materials, applications, modelling and performance improvement. Renew. Sust. Energy Rev..

[6] Shi X., Chen L., Uher C. (2016). Recent advances in high-performance bulk thermoelectric materials. Int. Mater. Rev..

[7] Shuai J., Mao J., Song S., Zhang Q., Chen G., Ren Z. (2017). Recent progress and future challenges on thermoelectric Zintl materials. Mater. Today Phys..

[8] Zhang Q.H., Huang X.Y., Bai S.Q., Shi X., Uher C., Chen L.D. (2016). Thermoelectric devices for power generation: Recent progress and future challenges. Adv. Eng. Mater..

[9] Wei J., Yang L., Ma Z., Song P., Zhang M., Ma J., Yang F., Wang X. (2020). Review of current high-ZT thermoelectric materials. J. Mater. Sci..

[10] Hu L., Wu H., Zhu T., Fu C., He J., Ying P., Zhao X. (2015). Tuning multiscale microstructures to enhance thermoelectric performance of n-type Bismuth-Telluride-based solid solutions. Adv. Energy Mater..

[11] Liu W., Wang H., Wang L., Wang X., Joshi G., Chen G., Ren Z. (2013). Understanding of the contact of nanostructured thermoelectric n-type Bi_2_Te_2.7_Se_0.3_ legs for power generation applications. J. Mater. Chem. A.

[12] Mao J., Zhu H., Ding Z., Liu Z., Gamage G.A., Chen G., Ren Z. (2019). High thermoelectric cooling performance of n-type Mg_3_Bi_2_-based materials. Science.

[13] Tamaki H., Sato H.K., Kanno T. (2016). Isotropic conduction network and defect chemistry in Mg^3+^
*δ*Sb_2_-based layered zintl compounds with high thermoelectric performance. Adv. Mater..

[14] Zhang J., Song L., Pedersen S.H., Yin H., Hung L.T., Iversen B.B. (2017). Discovery of high-performance low-cost n-type Mg_3_Sb_2_-based thermoelectric materials with multivalley conduction bands. Nat. Commun..

[15] Zhang J., Song L., Iversen B.B. (2019). Insights into the design of thermoelectric Mg_3_Sb_2_ and its analogs by combining theory and experiment. NPJ Comput. Mater..

[16] Shi X., Wang X., Li W., Pei Y. (2018). Advances in thermoelectric Mg_3_Sb_2_ and its derivatives. Small Methods.

[17] Zhou Z., Han G., Lu X., Wang G., Zhou X. (2022). High-performance magnesium-based thermoelectric materials: Progress and challenges. J. Magnes. Alloys.

[18] Shang H., Liang Z., Xu C., Mao J., Gu H., Ding F., Ren Z. (2020). N-Type Mg_3_Sb_2-x_Bi_x_ alloys as promising thermoelectric materials. Research.

[19] Chen Y., Wang C., Ma Z., Li L., Li S., Wang J. (2021). Improved thermoelectric performance of n-type Mg_3_Sb_2_–Mg_3_Bi_2_ alloy with Co element doping. Curr. Appl. Phys..

[20] Jiang F., Feng T., Zhu Y., Han Z., Shu R., Chen C., Zhang Y., Xia C., Wu X., Yu H. (2022). Extraordinary thermoelectric performance, thermal stability and mechanical properties of n-type Mg_3_Sb_1.5_Bi_0.5_ through multi-dopants at interstitial site. Mater. Today Phys..

[21] Shang H., Liang Z., Xu C., Song S., Huang D., Gu H., Mao J., Ren Z., Ding F. (2020). Ntype Mg_3_Sb_2-x_Bi_x_ with improved thermal stability for thermoelectric power generation. Acta Mater..

[22] Yang J., Li G., Zhu H., Chen N., Lu T., Gao J., Guo L., Xiang J., Sun P., Yao Y. (2022). Next-generation thermoelectric cooling modules based on high-performance Mg_3_(Bi, Sb)_2_ material. Joule.

[23] Peng Q., Yuan X., Zhao S., Zhou Y., Wen X., Chen X.-j. (2023). Active-learning search for unitcell structures: A case study on Mg_3_Bi_2-x_Sb_x_. Comput. Mater. Sci..

[24] Peng Q., Zhao S., Yuan X., Chen X.-J. (2022). Elasticity of Mg_3_Bi_2-x_Sb_x_. Materials.

[25] Huang B., Luo P., Li Z., Liu X., Zhang Y., Tang Y., Xing J., Zhang J., Guo K., Dong Z. (2023). Improving Thermoelectric Performance of n-Type Mg_3_Bi_2_-Based Materials by Introducing a Spatially Confined Magnetic Ordered Structure. ACS Appl. Energy Mater..

[26] Zhang Y.-b., Liang J.-S., Liu C., Zhou Q., Xu Z., Chen H.-b., Li F.-c., Peng Y., Miao L. (2024). Enhancing thermoelectric performance in P-Type Mg_3_Sb_2_-based zintls through optimization of band gap structure and nanostructuring. J. Mater. Sci. Technol..

[27] Witkoske E., Wang X., Maassen J., Lundstrom M. (2019). Universal behavior of the thermoelectric figure of merit, zT, vs. quality factor. Mater. Today Phys..

[28] Chen K.-X., Li M.-S., Mo D.-C., Lyu S.-S. (2018). Nanostructural thermoelectric materials and their performance. Front. Energy.

[29] Biswas K., He J., Blum I.D., Wu C.-I., Hogan T.P., Seidman D.N., Dravid V.P., Kanatzidis M.G. (2012). High-performance bulk thermoelectrics with all-scale hierarchical architectures. Nature.

[30] Nakamura Y., Isogawa M., Ueda T., Yamasaka S., Matsui H., Kikkawa J., Ikeuchi S., Oyake T., Hori T., Shiomi J. (2015). Anomalous reduction of thermal conductivity in coherent nanocrystal architecture for silicon thermoelectric material. Nano Energy.

[31] Zhang Q., He J., Zhu T., Zhang S., Zhao X., Tritt T.M. (2008). High figures of merit and natural nanostructures in Mg_2_Si_0.4_Sn_0.6_ based thermoelectric materials. Appl. Phys. Lett..

[32] Mo X., Liao J., Yuan G., Zhu S., Lei X., Huang L., Zhang Q., Wang C., Ren Z. (2022). High thermoelectric performance at room temperature of n-type Mg_3_Bi_2_-based materials by Se doping. J. Magnes. Alloys.

[33] Knura R., Parashchuk T., Yoshiasa A., Wojciechowski K.T. (2021). Origins of low lattice thermal conductivity of Pb_1-x_Sn_x_ Te alloys for thermoelectric applications. Dalton Trans..

[34] Ding J., Lanigan-Atkins T., Calderón-Cueva M., Banerjee A., Abernathy D.L., Said A., Zevalkink A., Delaire O. (2021). Soft anharmonic phonons and ultralow thermal conductivity in Mg_3_(Sb,Bi)_2_ thermoelectrics. Sci. Adv..

[35] Peng W., Petretto G., Rignanese G.-M., Hautier G., Zevalkink A. (2018). An unlikely route to low lattice thermal conductivity: Small atoms in a simple layered structure. Joule.

[36] Zhu Y., Xia Y., Wang Y., Sheng Y., Yang J., Fu C., Li A., Zhu T., Luo J., Wolverton C. (2020). Violation of the T- 1 relationship in the lattice thermal conductivity of Mg_3_Sb_2_ with locally asymmetric vibrations. Research.

[37] Han Z., Gui Z., Zhu Y., Qin P., Zhang B.-P., Zhang W., Huang L., Liu W. (2020). The electronic transport channel protection and tuning in real space to boost the thermoelectric performance of Mg^3+^
*δ*Sb_2-y_Bi_y_ near room temperature. Research.

[38] Imasato K., Kang S.D., Snyder G.J. (2019). Exceptional thermoelectric performance in Mg_3_Sb_0.6_Bi_1.4_ for low-grade waste heat recovery. Energy Environ. Sci..

[39] Shi X., Zhang X., Ganose A., Park J., Sun C., Chen Z., Lin S., Li W., Jain A., Pei Y. (2021). Compromise between band structure and phonon scattering in efficient nMg_3_SbBi_2-x_Bi_x_ thermoelectrics. Mater. Today Phys..

[40] Xu C., Liang Z., Shang H., Wang D., Wang H., Ding F., Mao J., Ren Z. (2021). Scalable synthesis of n-type Mg_3_SbBi_2-x_Bi_x_ for thermoelectric applications. Mater. Today Phys..

[41] Imasato K., Wood M., Anand S., Kuo J.J., Snyder G.J. (2022). Understanding the high thermoelectric performance of Mg_3_Sb_2_-Mg_3_Bi_2_ alloys. Adv. Energy Sustain. Res..

[42] Pan Y., Yao M., Hong X., Zhu Y., Fan F., Imasato K., He Y., Hess C., Fink J., Yang J. (2020). Mg_3_(Bi, Sb)_2_ single crystals towards high thermoelectric performance. Energy Environ. Sci..

[43] Togo A., Chaput L., Tanaka I. (2015). Distributions of phonon lifetimes in Brillouin zones. Phys. Rev. B.

[44] Li W., Carrete J., Katcho N.A., Mingo N. (2014). ShengBTE: A solver of the Boltzmann transport equation for phonons. Comput. Phys. Commun..

[45] Korotaev P., Shapeev A. (2020). Lattice dynamics of Yb_x_Co_4_Sb_12_ skutterudite by machine learning interatomic potentials: Effect of filler concentration and disorder. Phys. Rev. B.

[46] Verdi C., Karsai F., Liu P., Jinnouchi R., Kresse G. (2021). Thermal transport and phase transitions of zirconia by on-the-fly machine-learned interatomic potentials. NPJ Comput. Mater..

[47] Li R., Liu Z., Rohskopf A., Gordiz K., Henry A., Lee E., Luo T. (2020). A deep neural network interatomic potential for studying thermal conductivity of *β*-Ga_2_O_3_. Appl. Phys. Lett..

[48] George J., Hautier G., Bartók A.P., Csányi G., Deringer V.L. (2020). Combining phonon accuracy with high transferability in Gaussian approximation potential models. J. Chem. Phys..

[49] Eriksson F., Fransson E., Erhart P. (2019). The Hiphive Package for the extraction of high order force constants by machine learning. Adv. Theory Simul..

[50] Fransson E., Eriksson F., Erhart P. (2020). Efficient construction of linear models in materials modeling and applications to force constant expansions. NPJ Comput. Mater..

[51] Yang H., Zhu Y., Dong E., Wu Y., Yang J., Zhang W. (2021). Dual adaptive sampling and machine learning interatomic potentials for modeling materials with chemical bond hierarchy. Phys. Rev. B.

[52] Zuo Y., Chen C., Li X., Deng Z., Chen Y., Behler J., Csányi G., Shapeev A.V., Thompson A.P., Wood M.A. (2020). Performance and cost assessment of machine learning interatomic potentials. J. Phys. Chem. A.

[53] Shapeev A.V. (2016). Moment tensor potentials: A class of systematically improvable interatomic potentials. Multiscale Model. Simul..

[54] Ouyang P., Yuan M.-h., Tang P., Zhang Q., Liu S., Shuai J., Li X.-G. (2023). Atomic Local Ordering and Alloying Effects on the Mg_3_(Sb_1–x_Bi_x_)_2_ Thermoelectric Material. ACS Appl. Mater. Interfaces.

[55] Kresse G., Furthmüller J. (1996). Efficiency of ab-initio total energy calculations for metals and semiconductors using a plane-wave basis set. Comput. Mater. Sci..

[56] Kresse G., Furthmüller J. (1996). Efficient iterative schemes for ab initio total-energy calculations using a plane-wave basis set. Phys. Rev. B.

[57] Wu X., Vanderbilt D., Hamann D. (2005). Systematic treatment of displacements, strains, and electric fields in density-functional perturbation theory. Phys. Rev. B.

[58] Huang L.-F., Zeng Z. (2015). Roles of mass, structure, and bond strength in the phonon properties and lattice anharmonicity of single-layer Mo and W dichalcogenides. J. Phys. Chem. C.

[59] Togo A., Tanaka I. (2015). First principles phonon calculations in materials science. Scr. Mater..

[60] Ziman J.M. (2001). Electrons and Phonons: The Theory of Transport Phenomena in Solids.

[61] Omini M., Sparavigna A. (1995). An iterative approach to the phonon Boltzmann equation in the theory of thermal conductivity. Phys. B Condens. Matter.

[62] Perdew J.P., Burke K., Ernzerhof M. (1996). Generalized gradient approximation made simple. Phys. Rev. Lett..

[63] Wang V., Xu N., Liu J.-C., Tang G., Geng W.-T. (2021). VASPKIT: A user-friendly interface facilitating high-throughput computing and analysis using VASP code. Comput. Phys. Commun..

[64] Jain A., Ong S.P., Hautier G., Chen W., Richards W.D., Dacek S., Cholia S., Gunter D., Skinner D., Ceder G. (2013). Commentary: The Materials Project: A materials genome approach to accelerating materials innovation. APL Mater..

[65] Gladishevskij E., Kripjakevic P., Bodak O. (1967). The crystal structures of the compound CaAl_2_Si_2_ and its analogues. Ukr. Fiz. Zh..

[66] Shuai J., Wang Y., Kim H.S., Liu Z., Sun J., Chen S., Sui J., Ren Z. (2015). Thermoelectric properties of Na-doped Zintl compound: Mg_3-x_Na_x_Sb_2_. Acta Mater..

