# Supplementary material for: Lattice Thermal Conductivity of Mg3(Bi,Sb)2 Nanocomposites: A First-Principles Study
Source: Nanomaterials (Basel). 2023 Nov 13;13(22):2938. doi: 10.3390/nano13222938 (PMC10675190; doi:10.3390/nano13222938)
Supplement: Supplementary file 1 [file nanomaterials-13-02938-s001.zip › nanomaterials-2671842-supplementary.pdf]

Supplementary Information

Lattice Thermal Conductivity of $Mg_3(Bi,Sb)_2$ Nanocomposites: a First-Principles Study

Qing Peng [1,2,3,*,$], Xiaoze Yuan [1,$], Shuai Zhao [1,4] and Xiao-jia Chen [2,*]

1. School of Science, Harbin Institute of Technology, Shenzhen 518055, China
2. The State Key Laboratory of Nonlinear Mechanics, Institute of Mechanics, Chinese Academy of Sciences, Beijing, 100190, P. R. China
3. Guangdong Aerospace Research Academy, Guangzhou 511458, China
4. Department of Modern Mechanics, University of Science and Technology of China, Hefei, Anhui 230026, P. R. China

$ These authors contributed equally to this work.
* Correspondences: pengqing@imech.ac.cn (Q.P.), xjchen@hit.edu.cn (X.C.)

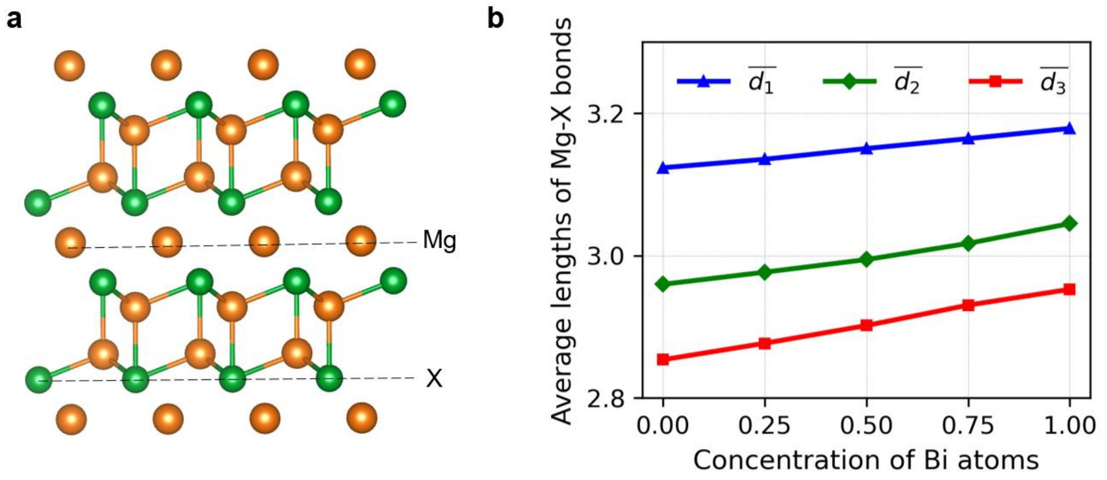

Figure S1. (a) Crystal structure model of Mg_3X_2 (X = Sb, Bi); (b) The average lengths of Mg-X ionic bonds ($\overline{d_1}$), vertical Mg-X covalent bonds ($\overline{d_2}$), and three symmetry-equivalent tilted covalent Mg-X bonds ($\overline{d_3}$).

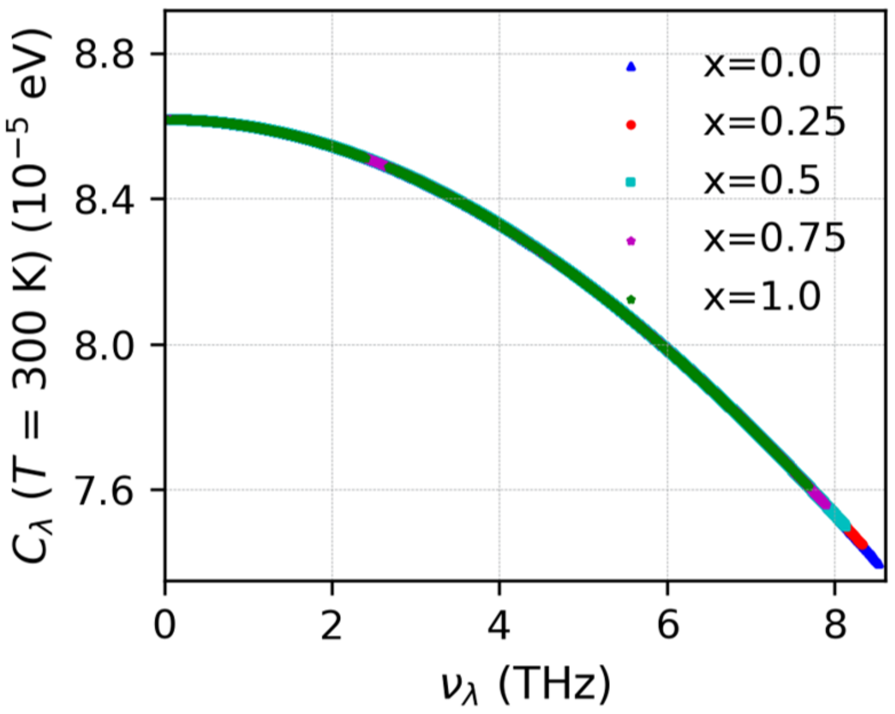

Figure S2. The heat capacity of $Mg_3(Bi_xSb_{1-x})_2$ (0≤x≤1) at 300K.
